# The Regulation of Energy Intake in Infancy: A Narrative Review

**DOI:** 10.1007/s13679-025-00653-9

**Published:** 2025-07-25

**Authors:** Amanda K. Crandall, Lucy Loch, Ashley N. Gearhardt, Julie C. Lumeng

**Affiliations:** 1 Division of Developmental and Behavioral Pediatrics, Department of Pediatrics, University of Michigan, North Campus Research Complex, Building 520, Ann Arbor, MI 48109 USA; 2 Department of Psychology, University of Michigan, Ann Arbor, MI USA

**Keywords:** Infant energy intake, Infant feeding behavior, Infant appetite, Infant homeostatic eating, Infant reward-driven eating, Caloric compensation

## Abstract

**Purpose of the Review:**

To examine evidence for factors that program and impact regulation of energy intake in infancy.

**Recent Findings:**

Infants regulate energy intake within a narrow margin, and this capacity may degrade with age as volume-based regulation emerges. However, feeding frequency, milk/formula protein content and/or structure, portion size, and caregiver encouragement can interrupt this regulation. Emerging evidence also suggests that some infants exhibit signs of reward-driven eating, which may also affect energy intake.

**Summary:**

Despite emphasis on obesity prevention in infancy, few studies directly examine milk/formula, food, or energy intake and even fewer use experimental methods to assess causation. Existing experimental evidence suggests a limited and diminishing regulation of energy intake through infancy and beyond. More research is needed to understand individual differences between infants in regulation of energy intake and propensity for reward-driven eating.

## Introduction

**Fig. 1:**
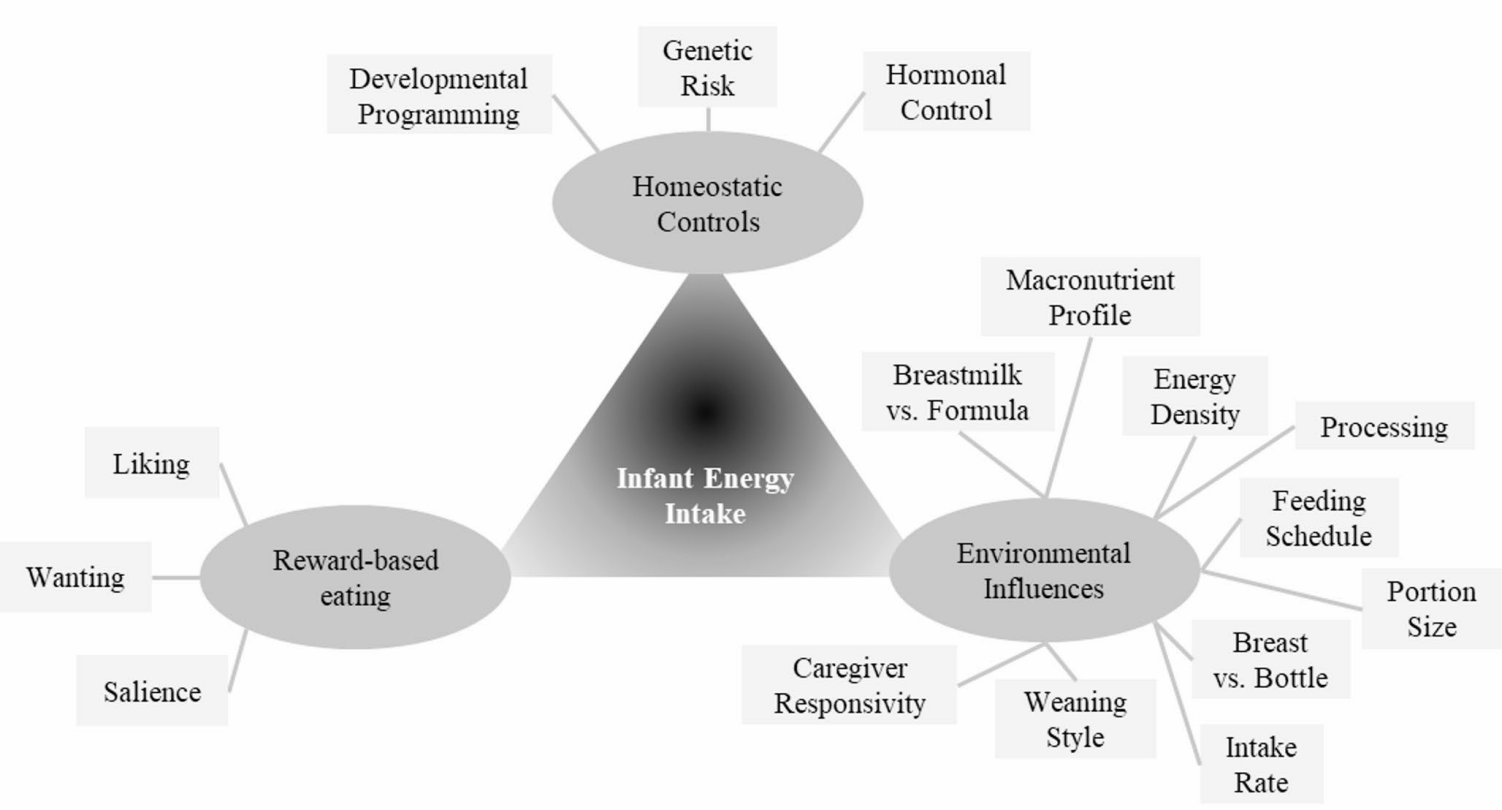
Conceptual model of the domains of control over infant energy intake

Early research on infant energy intake largely focused on understanding normal infant growth patterns to prevent growth faltering and improve survival [1–3]. Through the twentieth century, as infant survival rates increased and women sought alternatives to breastfeeding, more research focused on improving infant formula, bottles, and nipples to optimize infant health and growth [4, 5]. In the 1950s, with a growing focus on the importance of the maternal-infant interaction to infant development, pediatric guidelines were updated to the current recommendation to feed on demand instead of on a schedule. Healthy full-term infants are, at least to a certain extent, opportunistic feeders. Therefore, when infants feed on demand as opposed to on a schedule, they feed more frequently, increase energy intake, and gain more weight [1, 6–9]. As survival of premature infants increased through the 1980s, the research focused on strategies to increase energy intake to promote growth expanded further to focus on this population and their unique challenges [10–12]. The rise of childhood obesity in the 1990s renewed research interest in normal growth patterns of full-term infants, but newly considered the potential of infants to overeat [13–16]. Research therefore began to emerge on interventions to prevent overeating and excess weight gain, including feeding responsively [17, 18], delaying solid food introduction [19], improving infant flavor acceptance through early exposure [20, 21], and limiting sugar-sweetened beverages [22]. However, more research is needed to understand mechanisms of infant obesity risk. Behavioral susceptibility theory posits that some children are genetically predisposed to a greater appetitive drive and are more sensitive to the “obesogenic” environment [23]. This theory has prompted investigation into appetitive characteristics measurable in infancy, to identify those most at risk. The current review examines the recent literature surrounding regulation of energy intake in infancy, with a focus on homeostatic- and reward-driven eating, and environmental influences on both (Fig. 1).

## Homeostatic Control of Energy Intake

The rapid increase in overweight and obesity over the past four decades [24–26] has reinforced the need to understand how genetic risk may promote energy intake in the current food environment. Genetic foundations for obesity are well-established [27–29]. Parent and child eating behaviors are strongly associated [30, 31], even in infancy [32, 33]. Homeostatic need is the primary driver of appetite [34, 35], but most animals’ energy intake exceeds immediate energy needs, storing energy in adipose tissue for use in times of scarcity [36]. Nonetheless, eating behaviors, including appetitive drive, show individual differences even in infancy. Infant appetite and eating behavior are most often measured via parental report using the Baby Eating Behavior Questionnaire (BEBQ), which measures general infant appetite, enjoyment of food, food responsiveness, satiety responsiveness, and slowness in eating [37]. Differences in infant appetite, be they genetic, developmentally programmed during pregnancy, and/or apparent in hormonal controls, are measurable early in infancy and likely foundational to the next generation of interventions to prevent chronic disease.

### Genetic Origins and Developmental Programming

Although no studies have examined energy intake directly, some evidence suggests genetic contributors to energy intake are apparent in infancy. Maternal dietary restraint, which is associated with excess energy intake and obesity in adults, is associated with more liking-related facial expressions in response to sucrose in infants at age four months [33]. The Gemini Twin Cohort established genetic underpinnings for parental-reported infant appetitive characteristics [38], as measured by the BEBQ. Heritability was high in this cohort for food-avoidant behaviors, including slowness in eating and satiety responsiveness, and moderate for some food approach behaviors, including food responsiveness and enjoyment of food [38]. More recently, one small study showed that mutations of the leptin gene (i.e., chromosome 7q31.3, in humans) in healthy average-weight infants were associated with greater circulating leptin before age six months [39], which may be the first signs of leptin resistance (discussed below). Further, maternal-reported infant satiety responsiveness mediates the association between genetic risk for obesity and greater adiposity in the infant [40].

Infant eating behavior may also result from epigenetic changes and developmental programming during pregnancy. Greater maternal pre-pregnancy body mass index (BMI) and gestational weight gain are associated with greater infant food reinforcement, or food *wanting* [41]. Greater maternal pre-pregnancy BMI, gestational weight gain, and greater ultra-processed food intake during pregnancy predict lower maternal-reported infant satiety responsiveness [42, 43]. Among women with gestational diabetes, a self-reported diet rich in whole fruits and vegetables and minimally processed foods predicts later maternal-reported greater infant slowness in eating in their infants and less enjoyment of food among daughters [44]. Yet, paradoxically, maternal food preoccupation and responsiveness, assessed during pregnancy, are associated with greater maternal-reported infant satiety responsiveness at age six months [42]. Though based mainly on maternal reports, current evidence suggests a consistent association between maternal factors during pregnancy and infant appetitive characteristics.

There is also evidence of high food-approach appetitive characteristics measured directly from infant behavior starting very early in development. These are associated with parental-reported infant eating behavior, infant energy intake, and infant growth trajectories. The approach dimension of infant temperament is negatively associated with facial expressions of distaste and positively associated with food intake in the laboratory [45]. Greater infant distress during a brief mid-feeding delay is associated with greater milk/formula intake, and this association increases with age [46]. A vigorous sucking style at age four months predicts greater weight gain to age 12 months [47]. Parental-reported infant satiety responsiveness was negatively and robustly associated with the likelihood that an infant aged three to five months would accept an additional bottle after a standard meal, independent of infant size or parental feeding practices [48]. The consistency of evidence supports the theory of a combination of genetic influences and developmental programming of eating behavior that emerges very early in life.

### Hormonal Controls

Leptin, ghrelin, and adiponectin are important metabolic hormones regulating energy intake during infancy [49–51]. Leptin is primarily generated by white adipose tissue and regulates energy balance by inhibiting hunger signals and increasing energy expenditure [49]. Among adults and children, adiposity is positively associated with both leptin and leptin resistance [52, 53]. Neonatal leptin is positively associated with birthweight [54] and being large for gestational age [51, 55], and leptin in infancy is negatively associated with protein-energy malnutrition [56]. Cord blood leptin is negatively associated with weight-for-length-gain to age 12 months [57], suggesting a mechanism for catch-up growth. One recent cohort study showed a weak but consistent cross-sectional and prospective positive association between leptin and fat mass percentage in the first six months of infancy [58]. Thus, individual differences in leptin levels in infancy may be involved in the developmental programming of appetite and adiposity.

Adiponectin, like leptin, is a hormone secreted by adipose tissue. It was discovered in the late 1990s, and in adults and children is negatively associated with energy intake and positively associated with insulin sensitivity [59]. Adiponectin is greater in the neonatal period than in childhood [60], positively associated with birthweight, and lower among small for gestational age infants [60]. Cord blood adiponectin is also negatively associated with weight, weight gain, and BMI at age 12 months [61]. More research is needed to understand the role of adiponectin in growth and the development of adiposity.

Ghrelin is primarily produced by neuroendocrine cells in the gastrointestinal tract and stimulates appetite, countering the effects of leptin and adiponectin [53, 62]. As in adults and children, ghrelin in infants aged six months is positively associated with fasting time [58]. Among full-term infants, being small for gestational age predicts greater ghrelin at age three months [63], and emerging evidence suggests a role in promoting hunger to support catch-up growth [64], which is also a risk factor for later obesity [16, 65]. Among full-term infants with weight appropriate for gestational age, ghrelin is weakly positively associated with maternal-reported infant hunger frequency and adiposity at age six months [58]. Evidence suggests that ghrelin stimulates appetite in infants similarly to children and adults. Still, more research is needed to understand how ghrelin may change and adapt based on energy sources.

## Reward-Driven Eating

Some eating behaviors are hedonically- as opposed to homeostatically-driven. Reward-driven eating is eating for pleasure as opposed to hunger [66, 67]. It is hypothesized to be composed of three main components: *liking*,* wanting*, and *salience* [68]. *Liking* is the hedonic response that occurs while eating [68] and is associated with activation of the endogenous opioid system [66]. *Wanting* is the desire for food, which can manifest as motivation to seek food or the subjective experience of craving [68]. *Wanting* is strongly associated with activation of the mesolimbic dopamine system [68]. Finally, *salience* is the degree to which signals that confer the availability of a particular food (e.g., smell of French fries, image of a cookie, etc.) capture attention. Highly salient cues can also trigger activity in the mesolimbic dopamine system, thus motivating food-seeking behavior [68, 69]. These sub-components of reward are often intertwined, such that foods that are liked during intake are then more likely to be wanted, and the associated cues become more salient [68, 69].

### Liking

The BEBQ enjoyment of food subscale [37] may capture *liking* in infancy. A one-point increase in maternal-reported infant enjoyment of food is associated with a 0.26 standard deviation increase in infant weight at age nine months [70], and a 0.45 month delay in introduction of solid foods, presumably due to parents perceiving infants to enjoy milk or formula [71]. Exposure to sucrose can evoke an opioid response and trigger specific facial expressions such as lip smacking and tongue protrusions in both animal and human infants [72, 73]. Infants are born with a predisposition for liking sweet tastes, perhaps to enhance breastmilk intake, which is naturally sweet [21]. Neonates exhibit greater liking-related facial expressions in response to sucrose solution relative to water or bitter solutions [74]. Likewise, infants up to age six months will display three to five percent more *liking*-associated facial expressions for sucrose solutions relative to water [73]. Responses to sucrose have also been investigated in infant pain research. Sucrose administration is so effective at activating the endogenous opioid system implicated in liking that it is used as an analgesic during painful procedures (e.g., immunizations, newborn screens, etc.) [75]. A concentration of 24% sucrose, the same used in sucrose-liking response tasks, is effective at reducing pain in infancy [75]. The analgesic effects of sucrose in the neonate are positively associated with weight gain to age 18 months, particularly among those with higher birth weight-for-length [76]. Thus, sucrose’s capacity to engage the liking-related opioid system in infancy may predict risk for excess energy intake. Still, no studies to date have shown this effect directly.

### Wanting

Infant food responsiveness [37, 77] may capture *wanting* and *salience.* Food responsiveness tracks strongly from ages one to 10 months, suggesting a relatively stable phenotype [78]. Food responsiveness was also associated with infant growth and adiposity in a meta-analysis [79]. For example, one longitudinal study found that a one-point increase in food responsiveness at age three months was associated with a 0.24 z-score increase in BMI at age six months [80]. This association persisted as infants grew into toddlers, though the magnitude declined over time; by age 15 months, a one-point increase in food responsiveness at age three months predicted a 0.17 z-score increase in BMI [81]. Infant food responsiveness is also associated with energy intake. In one longitudinal study, the change in caloric compensation capacity between ages 11 and 15 months was negatively associated with food responsiveness at age 15 months [82]. In another study, food responsiveness among infants aged three to five months was positively associated with the likelihood of accepting an additional bottle after finishing their typical meal (i.e., an adapted eating in the absence of hunger task) [48].

An established behavioral measure of *wanting* food is the relative reinforcing value (RRV) of food task [83], in which participants click a button to earn points towards a food reward, providing a measure of motivation to work for food, or an index of food *wanting* [69]. In adults and children, willingness to work for food on an RRV task is positively associated with future energy intake and BMI [84]. This RRV task has been modified to be developmentally appropriate for infants aged nine months and older [41, 85]. In this version of the task, infants press a button to earn access to a preferred food or age-appropriate task (e.g., playing with a toy). The task stops when infants cry or display that they have lost interest [41, 86]. At ages nine to 18 months, the RRV of food is moderately positively associated with maternal-reported infant food responsiveness and general appetite, and overweight or obesity [41, 87]. No studies have shown an association between infant RRV of food and energy intake, but there is a positive association with weight-for-length z-scores over time [85]. Additionally, one randomized controlled trial (RCT) showed that infant food wanting, as measured by RRV of food, can be reduced through an intervention that includes repeated exposure to a non-food reward alternative (i.e., a music enrichment program) [88]. Thus, greater food *wanting* may be an important and malleable aspect of reward-driven eating in infancy that deserves further study.

### Salience

Food cue *salience* can be measured in infancy using a behavioral task where foods (e.g., cookies, strawberries, green beans) and non-food items (e.g., toys) are displayed to infants aged six to 12 months, and infant reactions to these items (i.e., reaching for, touching) are coded [89]. In general, infants react more to food cues than non-food cues, as indicated by purposeful touching [89]. Food cue salience in this task is positively associated with parental-reported infant food responsiveness and weight gain since birth [89]. Given that maternal factors have been associated with other measures of reward-driven eating in infants, more research is needed to investigate whether food cue salience is related to maternal characteristics (e.g., maternal BMI, maternal dietary restraint). Likewise, it is unknown if reward-driven eating is malleable throughout development or if it is a stable phenotype that must be managed.

## Environmental Influences

The environment in which infants eat is rapidly changing with advances in food science and a growing understanding of development. A greater ability to ethically manipulate environmental influences in an experimental setting has led to more research on this topic. Classic research in the early 1900s established that infants have the capacity to adjust intake of breastmilk and solid food to maintain approximately the same energy intake. This capacity for compensation, however, is incomplete [5, 90, 91]. For example, increasing the caloric density of solid foods does not reduce breastmilk intake among infants at high risk for growth faltering, suggesting limited capacity for caloric compensation in this high-risk context [92]. Yet, in a plentiful food environment, obesity prevalence has increased even in infancy [93, 94]. Environmental factors that may interact with behavioral susceptibility [95] to increase obesity risk in infancy include milk/formula composition [96, 97], feeding modality [98], and overfeeding by caregivers to prevent hunger [99] and/or encourage growth [100]. Unlike in children and adults, energy intake in infancy, especially in the first months after birth, is less likely to be governed by scheduled mealtimes. Though some infants may establish a feeding schedule, feeding frequency shows significant individual differences and bidirectional associations with energy intake [101]. Feeding size has been positively associated with weight-for-length z-scores in later infancy and toddlerhood [101]. Finally, evidence suggests that caregiver responsivity to infant hunger and satiation cues predicts healthy regulation of energy intake and mitigates obesity risk [102, 103]. Research continues to advance understanding of these constructs and elucidate the importance of tracking changes across developmental phases, between energy sources, and within the context of individual risk factors.

### Energy Source and Composition

Despite a significant body of research examining breastmilk [104] and formula [105] composition, few studies have tested effects of manipulating milk/formula composition on infant energy intake. Breastmilk contains leptin, adiponectin, ghrelin, and other metabolic hormones, while formula does not [58]. Despite a positive association between breastmilk leptin and breastfeeding infants’ serum leptin [106], ghrelin and leptin are greater in exclusively formula-fed, compared to exclusively breastfed, infants [58, 107]. Conversely, other studies have not detected a significant difference in leptin based on type of feeding [50]. On average, infants who are exclusively formula-fed, compared to those who are exclusively breastfed, have a 0.3 standard deviation unit lower maternal-reported enjoyment of food and 0.5 standard deviation unit lower food responsiveness at age three months, though these differences were no longer significant by age 12 months [43]. In the study of food cue salience described earlier, breastfeeding was the most robust predictor identified, and showed a negative association [89]. Despite these patterns, it is important to recognize that there remains significant intragroup variability in eating behavior among both breast- and formula-fed infants.

While formula macronutrient composition varies across brands, there is otherwise little variability. In contrast, breastmilk macronutrient composition varies significantly, both between and within mothers [58, 96, 98]. Breastmilk fat content and intake are cross-sectionally and prospectively positively associated with infant adiposity [96, 108]. In one study of breastfed infants, early infancy breastmilk fat content was positively associated with later infancy adiposity, while protein content was negatively associated [96]. Further, breastmilk energy density was positively associated with later infancy adiposity and earlier satiation and negatively associated with feeding frequency [96]. Adiponectin is detectable in breastmilk at birth, increases until age one month, and on average shows little change thereafter [108]. Although breastmilk adiponectin is prospectively positively associated with overweight risk by age two years [109], more recent evidence has shown no association of breastmilk adiponectin with infant body size or composition [108]. A recent study showed that reducing maternal energy intake reduced breastmilk leptin and adiponectin, but infant milk intake and growth rate did not significantly change over the two week study period [110]. Although the animal literature suggests that metabolic hormones in breastmilk, such as obestatin and growth hormones, may influence the development of human infant metabolism and disease risk, current evidence is lacking in human infants [104, 111]. More research is needed to understand how breastmilk may transmit risk or protective factors for the development of infant appetite. The largest RCT of breastfeeding to date, however, did not detect a significant effect of breast- compared to formula-feeding on weight gain or growth among healthy, full-term infants [112]. Thus, the associations described above are likely not causal, and there may be unmeasured confounding that increases the likelihood of formula feeding, excess energy intake, and adiposity and rapid growth.

### Energy Density, Macronutrient Profile, and Processing in the Infant Diet

Recent research has attempted to understand the limits, directionality, and developmental trajectory of caloric compensation in infancy. One RCT involving infants younger than age two months tested the effect of reducing the energy and protein content of formula. According to parents’ intake reports, infants who received the experimental formula consumed more across the four-month study period, effectively upregulating intake and consuming the same amount of energy as the control group. This caloric compensation continued when solid foods were introduced [105]. Likewise, a small RCT among infants aged six to 10 months showed that increasing solid food energy density reduced intake, effectively down-regulating energy intake [113]. Conversely, infants aged four months in two within-subjects experiments testing the effect of an extensive protein hydrolysate formula [114] or cow’s milk formula supplemented with free-floating glutamate [114, 115], satiated at a lower volume (i.e., a reduction of approximately 15 to 18 mLs per feeding) and thereby reduced energy intake, which they did not compensate for at the subsequent feeding [114]. These findings align with those of a previous RCT showing that lower protein formula results in growth patterns resembling those of breastfed infants [116]. Another RCT with infants aged 11 months used an adapted caloric compensation protocol to examine energy intake after a high-energy density preload of pureed vegetables. Although infants decreased volume of food intake after the high-energy-density preload, they failed to fully down-regulate energy intake, consuming on average 44% more energy [117]. Overall, infants appear to compensate quite well for changes in energy density in milk/formula, but the nature of the protein structure in a formula may interrupt this caloric compensation. It is unknown if such differences in protein content of breastmilk may have the same effect. Reducing energy intake among infants in environments with plentiful resources may be beneficial. Indeed, formulas with hydrolyzed proteins have been shown to change growth patterns such that they are more similar to those of exclusively breastfed infants [118].

The level of processing of foods in the infant diet is an understudied contributor to energy intake. Ultra-processed foods (UPFs; industrially manufactured food products with minimal whole foods) now dominate the modern food supply and are a significant source of refined carbohydrates, saturated fats, and sodium [119, 120]. Such foods carry the risk of increasing energy intake [121]. Common UPFs with hyperpalatable ingredient combinations (e.g., sweets, salty snacks) are more likely to trigger cravings and are more often consumed to excess [122, 123]. Dietary trends show UPFs are becoming staples of infant diets [124, 125], and the effects of this change are not yet known. More research is needed to investigate how infants may differ in their susceptibility to exposure to highly rewarding UPFs in a manner that contributes to increased reward-driven eating, poor energy regulation, and excessive weight gain.

### Feeding Schedule and Portion Size

Feeding schedules and sizes vary significantly between infants. Parental-reported feeding/eating frequency is negatively associated with meal size throughout infancy and toddlerhood [126] and portion size is negatively associated with energy density in infants aged four to 11 months but not in toddlers [126]. Bottle size at infant age two months is also positively associated with formula intake per day, suggesting a portion size effect [127]. Volume of formula intake was positively associated with overweight at age 12 months; the greater risk of overweight among formula- compared to breast-fed infants was mitigated when formula-fed infant were fed smaller volumes [128]. Infants’ capacity for caloric compensation suggests that following a larger meal, infants will compensate by feeding less frequently (i.e., show greater satiety) or down-regulating intake at a subsequent meal. However, one recent within-subjects experiment showed that when infants were fed more frequently, energy intake increased by 5.2 kcals/kg over the six-hour study timeframe. This difference did not change over the 12 months of infancy [129]. The evidence across studies suggests a developmental progression of regulation of energy intake. The study noted earlier showed that when the energy density of solids is increased, infants aged 11 months do not significantly alter their volume of intake, and this behavior becomes more apparent at age 15 months [117]. Thus, infants regulate energy intake within a small margin in response to changes in energy density, but do not do so to the same extent in response to energy offered more often or in larger portions. Infants appear to shift from this energy-based intake regulation to the volumetric regulation characteristic of child and adult eating styles by about age two years [117, 130, 131]. The direct, experimental evidence for infant energy, schedule, and volume adjustment is sparse and disparate, and more research is needed to understand how infants respond to subtle changes in feeding frequency and portion size across more extended periods (e.g., days versus hours), between developmental stages (e.g., early infancy versus later infancy), and among infants with differing behavioral susceptibility.

### Feeding Modality and Speed

Recent evidence supports the historical understanding that differences in feeding modality, including breast versus bottle [132, 133] and nipple flow rate [134–136] can influence infant energy intake [137]. Many mothers report that their exclusively breastfed infants will refuse to bottle-feed, causing missed feedings [138]. At present, it is unknown if bottle refusal is due to infant preference or limited capacity to coordinate sucking from an artificial nipple [139]. Concomitant with feeding modality is the rate at which energy, particularly energy from milk/formula, is consumed. When offered milk/formula from a smaller nipple aperture, effectively slowing milk delivery, infants aged two to four months decrease milk intake in a single feeding [140]. The amount of time spent eating is strongly associated with milk intake [114]. Feeding directly from the breast, compared with a bottle, is associated with longer feeding duration in early infancy (i.e., 5.26 min longer at age one month), and this difference diminishes with age and disappears by age six months [141]. One cohort study showed a weak association between breastfeeding duration and maternal-reported infant slowness in eating at age 12 months [142]. Likewise, maternal-reported infant slowness in eating is associated with lower obesity risk [37, 143]. Reducing eating rate decreases energy intake in adults and children [144, 145]. Artificial nipple flow rates are highly variable, and the labeled size often does not correspond to actual nipple flow rate [146, 147]. Research has shown a positive association between bottle-feeding exposure, independent of breastmilk versus formula, and obesity risk in infancy [133] and later childhood [132, 148]. More research is needed to understand the mechanisms of this association and explore how eating rate may influence the development of eating behavior and obesity risk, particularly among infants with other risk factors.

### Communication with Caregiver

Parents are believed to strongly influence the development of energy intake regulation during infancy [149]. Although the capacity to regulate energy intake appears to decline across infancy, it remains more robust during childhood than in adulthood [150], and there are associations between obesity risk and both pressuring [151] and restrictive [152] parental feeding practices. Caregiver encouragement to eat is more apparent during infancy, likely due to infants’ more limited repertoire of strategies to resist eating and the emphasis on adequate growth during infancy for parents and pediatricians [153, 154]. The positive associations between bottle-feeding and obesity risk [132, 133, 148], as well as those between bottle-feeding and energy intake [126, 127], are, in part, explained by caregivers’ bottle-feeding behaviors. Bottle-feeding intensity is positively associated concurrently and prospectively with caregiver encouragement to finish the bottle [155]. One experiment showed that infant-led feeding, as defined by an experimenter trained in infant satiation cues, as opposed to maternal-led infant feeding, resulted in a 1.8 mL/kg reduction in milk/formula intake during a single bottle-feeding interaction [156]. In another experiment, mothers were given an opaque, weighted bottle, making monitoring intake challenging. The opaque, weighted bottle slowed milk/formula intake rate by 1.2 mL/min and reduced intake by 1.7 mL/kg [157]. When formula composition is varied experimentally in the hydrolyzed protein studies noted earlier, infant satiation cues occur at lower intake volumes, and whether the feeding was concluded depended on maternal responsiveness [115]. Evidence conflicts as to whether responsive feeding has differential effects on bottle-fed versus breastfed infants, but breastfeeding is thought to naturally encourage responsive feeding because the mother is less able to measure milk intake and must rely more on infant cues [17, 158, 159]. The study noted earlier in which infants’ capacity to compensate for an increase in energy density of pureed solids decreased with age, also showed that the magnitude of decline between ages 11 and 15 months was positively associated with parental sensitivity [82]. The preponderance of evidence suggests that among healthy, full-term infants, allowing the infant, as opposed to the caregiver, to choose when the feeding ends results in lower energy intake.

RCTs that include responsive feeding in infancy are effective at reducing obesity rates in infancy and beyond [17, 159, 160]. Conversely, one recent study showed that restrictive parenting practices, when applied to infants with greater parent-reported general appetites, were positively associated with a healthier weight trajectory across infancy [161]. Thus, although responsive feeding prevents excess energy intake in the average infant, more research is needed to understand if this remains true among infants with greater behavioral susceptibility for excess energy intake. It is also not yet known the extent to which responsive feeding reduces obesity risk by reducing energy intake directly during critical periods of adipose tissue development [162] versus, or in concert with, the fostering of a responsive and better-regulated eating style across development [163].

### Solid Food Introduction

The recommended age of solid food introduction was updated in the 1990s to prevent early introduction, thereby reducing risk for rapid weight gain and obesity [164]. Baby-led weaning is a modern trend in solid food introduction that has gained popularity over the past two decades [165]. Exact definitions vary, but these approaches typically encourage breastfeeding and allowing infants to self-feed non-pureed solid foods at their own pace. Cross-sectional evidence suggests that infants following a baby-led weaning diet consume more energy from milk/formula and less from solid foods, resulting in a diet with a greater proportion of energy from fat [166, 167]. Yet, cross-sectional evidence conflicts on energy intake, with one study showing no significant difference [168] and another showing a short-term reduction in energy intake [169]. RCTs examining the effects of baby-led weaning approaches have shown no difference in energy intake [170, 171], macronutrient profile [170, 171], or growth trajectories [172]. One exception is an RCT with infants aged five to six months showing that baby-led weaning significantly reduced weight at 12 months by 0.7 kg compared to traditional weaning methods [173]. Small effects on parental-reported infant appetite have been noted, with one RCT showing a small reduction in food fussiness and an increase in enjoyment of food at age 12 months, along with a counterintuitive reduction in satiety responsiveness at age 24 months among infants assigned to the baby-led weaning group [172]. Baby-led weaning appears safe, and current evidence suggests that the effect size on obesity risk and eating behavior development is small to none. This may be due to the propensity for infants to compensate for differences in available energy. More research is needed to understand if particular weaning styles may be better suited for some infants and families than others based on behavioral susceptibility.

## Conclusion and Future Directions

Historical evidence showed that lowering formula energy content caused infants to up-regulate intake but still consume less energy and gain less weight [5]. More recent evidence suggests more precise caloric compensation among infants than these earlier studies [113, 117], but also tested much smaller changes in energy [5, 91]. Taken together, this suggests that infants up- and down-regulate milk, formula, and solid food intake in response to changes in energy density only to a point, after which they are unable to compensate and/or their caregivers may intervene. This caloric compensation occurs in both directions, but there is much more evidence for infants up-regulating [5, 90, 91, 105] than down-regulating [113, 117] energy intake and caloric compensation for solid foods may be more difficult than for liquid calories (i.e., milk/formula) [117]. Likewise, infants exhibit this tight regulation of energy intake only in response to changes in energy density, but only partially compensate for changes in feeding frequency and portion size [98, 127, 129, 174]. Given rising rates of infant obesity [15, 93, 175] and evidence that infants can overeat [127, 129], more research is needed to understand if the capacity for regulation of energy intake operates in the same way in both directions and between solid and liquid calories, as well as how it changes throughout development. Although associations of formula- and bottle-feedings with obesity risk are likely not causal [112], there is evidence for properties of both breastmilk and formula that impact energy intake, and more research is needed to advance the precision of feeding recommendations, to support a healthy growth trajectory for both kinds of nutrition sources.

In childhood, caloric compensation capacity degrades with age [176], and recent research in infancy suggests that this decline begins even earlier in development [117]. A volumetric regulation of energy intake may emerge with age when the infant no longer relies solely on liquid calories [117]. Food consistency, speed of intake, and processing methods may have differential impacts depending on how sensitive this transition period proves to be for regulation of energy intake. Also missing from this research is the behavioral susceptibility between infants when comparing the regulation of energy intake. Multiple methods of assessing reward-driven eating in infancy have been developed and can be used to measure *liking*, *wanting*, and *salience.* Future research should examine these constructs and how they interact with homeostatic and environmental influences on hunger and satiation to better understand their dynamic interplay in infant energy intake.

## Data Availability

No datasets were generated or analysed during the current study.

## Abbreviations

BMI: Body mass index
BEBQ: Baby eating behavior questionnaire
RRV: Relative reinforcing value
UPF: Ultra-processed foods
RCT: Randomized controlled trial
